# Towards unified quality verification of synthetic count data with *countsimQC*

**DOI:** 10.1093/bioinformatics/btx631

**Published:** 2017-10-04

**Authors:** Charlotte Soneson, Mark D Robinson

**Affiliations:** 1 Institute of Molecular Life Sciences, University of Zurich, Zurich, Switzerland; 2 SIB Swiss Institute of Bioinformatics, Zurich, Switzerland

## Abstract

**Summary:**

Statistical tools for biological data analysis are often evaluated using synthetic data, designed to mimic the features of a specific type of experimental data. The generalizability of such evaluations depends on how well the synthetic data reproduce the main characteristics of the experimental data, and we argue that an assessment of this similarity should accompany any synthetic dataset used for method evaluation. We describe *countsimQC*, which provides a straightforward way to generate a stand-alone report that shows the main characteristics of (e.g. RNA-seq) count data and can be provided alongside a publication as verification of the appropriateness of any utilized synthetic data.

**Availability and implementation:**

*countsimQC* is implemented as an R package (for R versions ≥ 3.4) and is available from https://github.com/csoneson/countsimQC under a GPL (≥2) license.

## 1 Introduction

The analysis of biological data, for example those coming from high-throughput sequencing applications, is often highly dependent on sophisticated statistical tools and algorithms. Each such tool aims to extract a specific type of biologically relevant information from data, and its ability to perform the intended task is usually evaluated at the time of publication. Although the ultimate aim is to develop methods that give accurate results for real, experimental data, this is often difficult to assess in practice due to the lack of an independently validated ground truth. Thus, most statistical data analysis tools are (at least partly) evaluated on *synthetic* data, obtained either via parametric simulation or via sampling from experimental data. The synthetic data are assumed to share important characteristics with experimental data, but the signal of interest as well as other, potentially confounding, signals are completely controlled in the data generation. In such cases, the usefulness of any performance evaluation ultimately comes down to how realistic the synthetic data are. Thus, we argue that any evaluation relying on synthetic datasets should take care to verify that these reflect the most important characteristics of the data type of interest. Currently, many studies whose conclusions crucially depend on synthetic data do not provide extensive evidence of such similarity. We hypothesize that one reason for this is a lack of general guidelines regarding the set of aspects that should be investigated, as well as a straightforward way to easily generate a quality evaluation report for synthetic data, which exists for other aspects of high-throughput sequencing data analysis [e.g. FastQC (http://www.bioinformatics.babraham.ac.uk/projects/fastqc/), MultiQC (Ewels *et al.*, 2016)].

To fill this gap, we developed *countsimQC*, an R package that allows the user to quickly explore and compare a variety of characteristics across one or more count datasets and generate a summary report for evaluation and dissemination. For example, a user can visualize the characteristics of a synthetic dataset (e.g. a simulated RNA-seq count matrix) side by side with those of an experimental dataset of the type that it is intended to mimic. However, the package is not limited to such comparisons, and can be used to summarize and compare the properties of any collection of one or more count datasets.

## 2 Materials and methods

To run *countsimQC*, the user provides a count matrix and information about the experimental design for each dataset. The software then calculates a set of characteristics for each dataset and generates a stand-alone pdf or html report, which can be used to assess the properties of, and similarities between, the provided datasets. The evaluation criteria have been specifically selected to cover a broad range of important aspects of RNA-seq data and include the mean-dispersion relationship, library size distribution, expression distribution, and the fraction of zero counts per gene and sample as well as the relationship between these and a gene’s expression level or the total number of reads in a sample. The latter type of evaluation criteria is particularly useful for single-cell RNA-seq applications and other types of sparse count data. In addition to the graphical output, the *countsimQC* reports also contain a range of quantitative evaluation criteria comparing the distributions of the calculated statistics between datasets. The package is modular, and new evaluation criteria can easily be added by the user if desired. Moreover, the principles of *countsimQC* are not limited to count data, and given suitable quality assessment criteria the package can easily be extended to generate reports appropriate also for other data types.

## 3 Results

**Fig. 1:**
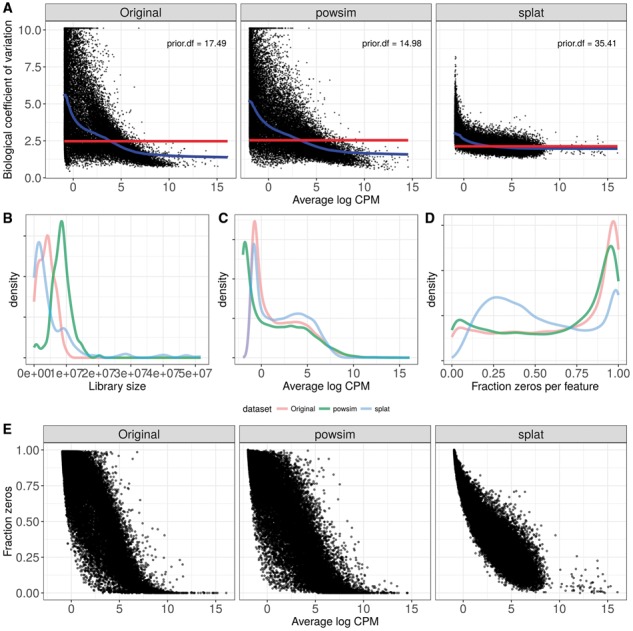
Example illustrations from a *countsimQC* report, comparing characteristics of three datasets: one real single-cell RNA-seq dataset (Original) and two datasets simulated using the real dataset as the underlying source of population parameters (powsim, splat). (**A**) BCV (biological coefficient of variation) as a function of average expression level, both calculated by edgeR (Robinson *et al.*, 2010). (**B**) Distribution of library sizes (total count sum) across all cells. (**C**) Distribution of average expression levels (log count per million, as calculated by edgeR) across all genes. (**D**) Distribution of the fraction of zeros across all genes. (**E**) Relationship between the average expression level (log count per million) and the fraction of zeros for all genes

To illustrate the output generated by *countsimQC*, we use two recently published R packages [the ’splat’ framework from splatter (v1.0.2) (Zappia *et al.*, 2017) and powsim (v1.0.1) (Vieth *et al.*, 2017)] to generate synthetic single-cell RNA-seq count matrices from an underlying, experimentally obtained count matrix [GSE48968-GPL13112 (Shalek *et al.*, 2014)], downloaded from the conquer repository [http://imlspenticton.uzh.ch:3838/conquer/ (Soneson and Robinson, 2017)]. Figure 1 shows a subset of the figures that are included in the *countsimQC* report. From these plots, it is clear that a single evaluation criterion is not enough to fully capture the degree of similarity between datasets, and that the simulation that is most similar to the underlying real data in one aspect can be far from it when another criterion is considered. Thus, having a comprehensive report illustrating the level of similarity from different aspects is useful not only for the synthetic data generator but also for the reader interested in judging the degree of generalizability of conclusions based on the synthetic data. The full report of the comparison summarized in Figure 1, as well as additional example reports for various types of real and synthetic count datasets can be accessed from https://github.com/csoneson/countsimQC.

## 4 Conclusions

With the *countsimQC* package, we wish to spark a discussion about the importance of evaluating the suitability of synthetic data. We encourage researchers who use synthetic data to draw conclusions to provide a report, such as that generated by *countsimQC*, as evidence that the synthetic data mimic real data, since this is not always easy to assess from a text description of a simulation model. We note that it is not the first effort in this direction; the compcodeR package (Soneson, 2014) as well as powsim (Vieth *et al.*, 2017) contain functions for summarizing the characteristics of a single dataset, and the splatter package (Zappia *et al.*, 2017) also provides the opportunity to generate figures comparing various aspects of a collection of single-cell RNA-seq datasets. However, to our knowledge it is the first independent, ‘one-stop’ solution to generate an extensive, publishable report comparing multiple count datasets, optionally including the code that was used to generate the plots.

There are of course situations where a high degree of similarity between the synthetic data and real data is less critical. For example, synthetic data are often used to illustrate the ability of algorithms and tools to correctly estimate hyperparameters of underlying data distributions, or to illustrate the influence of changing particular features of a dataset, possibly out of the realistic range, for better understanding of the advantages, disadvantages and sensitivities of the methodology. However, even in such situations we argue that it can be useful, both for the analyst and for the reader, to understand the characteristics of the generated data and how it compares to what would be seen in practice.

## Funding

This work was supported by the Forschungskredit of the University of Zurich [grant no. FK-16-107 to C.S.].

*Conflict of Interest*: none declared.
